# The Effect of Interferons on Presentation of Defective Ribosomal Products as HLA Peptides

**DOI:** 10.1016/j.mcpro.2021.100105

**Published:** 2021-06-01

**Authors:** Liran Komov, Dganit Melamed Kadosh, Eilon Barnea, Arie Admon

**Affiliations:** Faculty of Biology, Technion-Israel Institute of Technology, Haifa, Israel

**Keywords:** MHC peptidome, HLA peptidome, dynamic SILAC, interferon, defective ribosomal products–DRiPs, CCT, chaperonin-containing T-complex, DRiPs, defective ribosomal products, ER, endoplasmic reticulum, H/L, heavy-to-light, HLA, human leukocyte antigen, IFN, interferon, OSiPs, orphan subunit products, SILAC, stable isotope labeling by amino acids in cell culture, TAP, transporter associated with antigen processing

## Abstract

A subset of class I major histocompatibility complex (MHC)-bound peptides is produced from immature proteins that are rapidly degraded after synthesis. These defective ribosomal products (DRiPs) have been implicated in early alert of the immune system about impending infections. Interferons are important cytokines, produced in response to viral infection, that modulate cellular metabolism and gene expression patterns, increase the presentation of MHC molecules, and induce rapid degradation of proteins and cell-surface presentation of their derived MHC peptides, thereby contributing to the battle against pathogen infections. This study evaluated the role of interferons in the induction of rapid degradation of DRiPs to modulate the repertoire of DRiP-derived MHC peptides. Cultured human breast cancer cells were treated with interferons, and the rates of synthesis and degradation of cellular protein and their degradation products were determined by LC-MS/MS analysis, following the rates of incorporation of heavy stable isotope–labeled amino acids (dynamic stable isotope labeling by amino acids in cell culture, dynamic SILAC) at several time points after the interferon application. Large numbers of MHC peptides that incorporated the heavy amino acids faster than their source proteins indicated that DRiP peptides were abundant in the MHC peptidome; interferon treatment increased by about twofold their relative proportions in the peptidome. Such typical DRiP-derived MHC peptides were from the surplus subunits of the proteasome and ribosome, which are degraded because of the transition to immunoproteasomes and a new composition of ribosomes incorporating protein subunits that are induced by the interferon. We conclude that degradation of surplus subunits induced by the interferon is a major source for DRiP–MHC peptides, a phenomenon relevant to coping with viral infections, where a rapid presentation of MHC peptides derived from excess viral proteins may help alert the immune system about the impending infection.

## Highlights

• Degradation products of surplus subunits are often presented as HLA peptides.
• Interferons increase degradation and presentation of such defective products.
• Dynamic SILAC facilitates identification of such HLA peptides.
• This cellular pathway provides alert to the immune system about viral infections.

## Graphical Abstract

**Figure:**
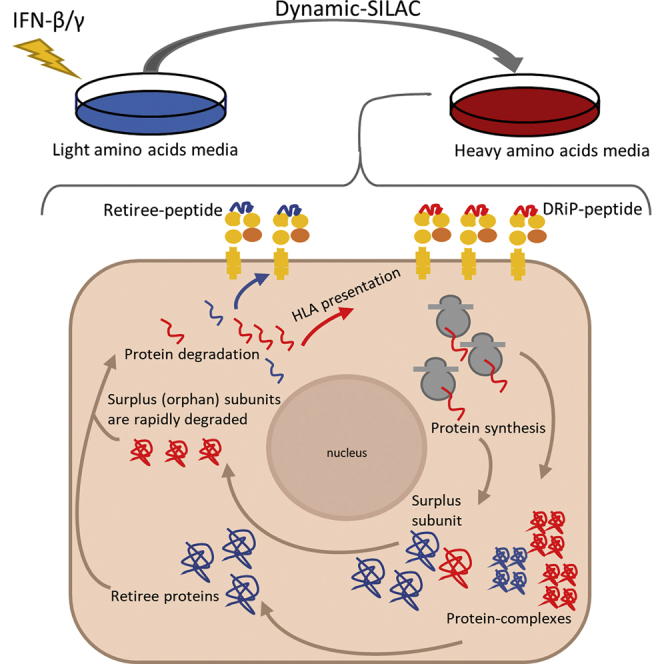

## In Brief

Surplus subunits of protein complexes are rapidly degraded, and some of their degradation products are shuttled for presentation by the MHC molecules at the cells’ surface. Interferons enhance synthesis and subsequent degradation of such surplus (orphaned) subunits, followed by presentation of resulting peptides by the MHC molecules. The phenomenon was followed in this study using the dynamic SILAC methodology, resulting in identification of many such defective ribosomal products, for example, from surplus subunits of the ribosomes and proteasomes.

The class I major histocompatibility complex (MHC), called in humans the human leukocyte antigen (HLA), informs the immune system about the ‘health state’ of the cells by transporting peptides from the interior of the cells and ‘presenting’ them at the cell surface. Detection by circulating T cells of MHC peptides derived from pathogens, or cancer proteins, triggers immune responses, leading to the elimination of the diseased cells and termination of the infection cycle and disease progression. The HLA class I molecules are encoded by HLA-A, HLA-B, and HLA-C, three highly polymorphic genes whose polymorphism strongly affects their peptide-binding properties, resulting in the presentation of significantly different peptides’ repertoires by each of the HLA allotypes (1, 2, 3, 4). The assortment of peptides presented by the MHC/HLA molecules on the cell surface is called the MHC or HLA peptidome, ligandome, or immunopeptidome, which are typically composed of tens of thousands of different peptides in each cell type. The peptides’ repertoire is shaped by the degradation scheme of cellular proteins, subsequent peptide transport into the endoplasmic reticulum (ER) *via* the transporter associated with antigen processing (TAP) (5, 6), peptide processing within the ER by resident proteases (7), chaperone activities, which load the peptides onto the MHC molecules (8), and the binding selectivity of each MHC allomorph (2, 3, 4). During pathogen infection, rapid degradation of pathogen proteins and presentation of derived peptides by the MHC are needed to alert the immune system of the impending infection and, subsequently, for the survival of the organism (9, 10). Viral proteins are often relatively stable, yet the infected cells need to degrade at least a fraction of them to present a sufficient number of these ‘nonself’ MHC peptides to stop the infection cycle (11, 12). To overcome the relative stability of viral proteins, the cells rapidly degrade immature cellular or viral proteins that do not assemble into the virions (called defective ribosomal products, DRiPs) (9, 13). In cultured cells, up to one-third of the MHC peptidome was proposed to derive from DRiPs (9, 13, 14, 15), which has subsequently been nicknamed ‘the DRiPome’ (14). DRiPs were suggested to derive from the initiation of translation from noncanonical sites (16, 17, 18), frameshifting (19, 20, 21, 22), oxidized and ubiquitinylated proteins induced by interferon (IFN)-γ (23), pioneer translation products (24), and translation of viral mRNA in the nucleus (25). However, it was also shown that full-sized, misassembled, surplus subunits of protein complexes contribute significantly to the HLA class I DRiPome in untreated cultured human cells (15). The degradation of excess subunits was shown to be essential for the proper function of the cells (26, 27). Nevertheless, the majority of the presented MHC class I peptides are thought to derive from normally degrading proteins, after completion of their functional lifetimes, referred to as retirees (28).

Analysis of virus-infected cells is the preferred approach for understanding the immunological relevance of the DRiPome. However, some viruses escape immune surveillance by altering and inhibiting the MHC-I presentation pipeline in infected cells (29), thus complicating the analysis of the virus-derived DRiPome because of the complex effect of the virus infection on the cells. Alternatively, parts of the cellular immune response that takes place during viral infection can be simulated by treating cells with IFNs, without infecting the cells. Cytokines, such as IFNs, are secreted from virus-infected cells and activated T cells, alert the immune system, and enhance the cellular response to the infection (30, 31). Therefore, studying the effect of IFNs on the HLA class I peptidome in cultured human cells may serve as a model for studying the effects of viral infection on the MHC peptidome and DRiPome.

The type I (IFN-α and IFN-β) and type II (IFN-γ) IFNs, investigated in this study, are key cytokines in the antiviral response. IFN-β is secreted from most cells in response to viral infection (32) including dendritic cells (33), and many other cell types in the body (34). At later stages of the immune response, IFN-γ is secreted from T cells and other immune cells (31). The IFNs strongly affect cellular metabolism and gene expression (35), including the transcription factors STAT1 and IRF-9 (36, 37), as well as MHC class I and class II molecules (36, 38, 39). The IFNs also upregulate antigen processing and presentation components, including proteases, such as ER aminopeptidase 1/2, and the TAP transporters (38, 40, 41, 42, 43, 44), as reviewed (2, 4, 7, 45). In addition, after IFN exposure, expression of the catalytic subunits of the immunoproteasome, that is, β1i/PSMB9, β2i/PSMB10, and β5i/PSMB8, are strongly induced, partially replacing the standard-proteasome catalytic subunits, β1/PSMB1, β2/PSMB2, and β5/PSMB5 (46, 47). After exposure to IFN, up to 60% of the standard proteasomes are thought to be replaced by the immunoproteasome (43). This replacement has been associated with an enhanced immune response, mediated by the production of peptides with hydrophobic or basic C termini, which are preferred substrates for the TAP transport, and possibly also better ligands for many of the MHC allotypes (48, 49). Thus, it was suggested that the effects of the IFNs on the immunopeptidome are largely mediated by the immunoproteasome (23, 45, 50, 51, 52, 53, 54, 55).

The effects of IFNs on the transcriptome (31), proteome (56, 57), and HLA-I peptidome (39, 58, 59, 60, 61, 62, 63) have been thoroughly studied. Also, the enhancement of MHC presentation by IFN treatment may add neoepitopes to the presented peptidome (55, 61, 64), this way inducing even stronger immune responses. However, a detailed analysis of the effect of IFN on the repertoires of peptides derived from DRiPs (the DRiPome) is still lacking. Most studies on the involvement of the DRiPome in virology monitored the dynamics of a few peptides (10) or cells infected with influenza or vaccinia virus (65, 66, 67). Dynamic stable isotope labeling by amino acids in cell culture (SILAC) followed by MS analysis was designed to follow dynamics of proteins turnover (dynamic SILAC) (68) and was used to distinguish between HLA peptides derived from DRiPs or retirees by simultaneously analyzing the kinetics of synthesis of the cellular proteins and their derived degradation products, the HLA peptides (15, 69, 70, 71, 72). Analysis of protein turnover kinetics revealed differential degradation rates for different proteins (73, 74, 75, 76, 77, 78). Using the dynamic SILAC approach, it is possible to distinguish between normal rapidly degrading proteins with short half-lives and DRiPs, which are HLA peptides produced from newly synthesized proteins before they mature, irrespective of their half-lives (15, 69, 71).

In this work, dynamic SILAC was exploited to determine whether DRiP processing rates are hastened to alarm the immune system and the antiviral defenses, of impending viral threat, simulated here by IFN-β and IFN-γ treatment.

## Experimental Procedures

### Cell Culture

The human breast cancer cell line, MCF-7 (obtained from the American Type Culture Collection), was maintained in Dulbecco's modified Eagle’s medium (DMEM), supplemented with 10% fetal calf serum, 1% L-glutamine, and 1% penicillin-streptomycin, in a humidified 5% CO_2_ incubator, at 37 °C. The endogenous HLA molecules of MCF-7 are HLA-A∗02:01, B∗18, B∗44, and C∗05, as determined by tissue typing performed in the Laboratory of Clinical Immunology and Tissue Typing, Rambam Hospital, Haifa.

### Dynamic SILAC Labeling With Heavy Isotope–Labeled Amino Acids (Leu+7, Arg+10, Lys+8) in IFN-Treated Cells

Cells (7 × 10^6^/25 ml) were plated in 150-mm petri dishes containing standard DMEM. The following day, the medium was supplemented with 500 U/ml IFN-β or 250 U/ml IFN-γ (PeproTech); control cells were left untreated. After 8 h of incubation, the medium was replaced with DMEM, lacking leucine, lysine, and arginine (Biological Industries), and supplemented 10% dialyzed fetal calf serum (Biological Industries) and heavy leucine (^13^C_6_,^15^N-Leu), heavy lysine (^13^C_6_,^15^N_2_-Lys), and heavy arginine (^13^C_6_,^15^N_4_-Arg) (Cambridge Isotope Laboratories) at final concentrations of 52, 147.6, and 87.3 mg/l, respectively, as well as with 500 U/ml IFN-β or 250 U/ml IFN-γ. After an additional 4-, 8-, or 12-h incubation with the heavy amino acid medium and the IFNs, the cells were harvested, counted, and solubilized with detergents, and HLA peptidomes and proteomes were analyzed, as described below.

### Experimental Design and Statistical Rationale

**Fig. 1:**
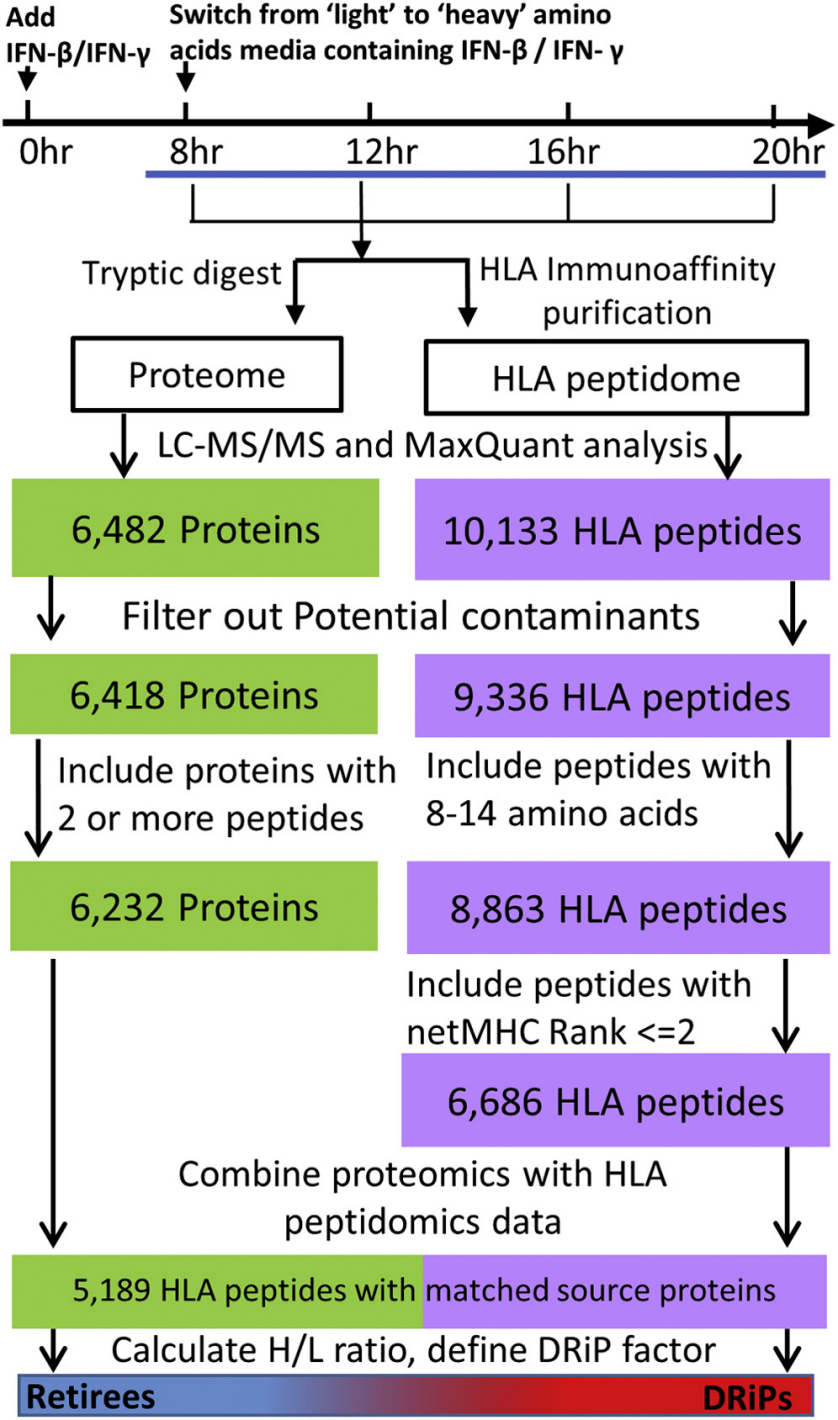
**Experimental flowchart of the dynamic SILAC samples.** Workflow for isolation and analysis of the proteome and the HLA class I peptidome of IFN-treated and untreated cells. HLA peptides were filtered, as indicated, to select the true HLA ligands; the filtered HLA peptidome and cellular proteome were matched to pair the HLA peptides with their source proteins. DRiP factors were calculated from the H/L ratios of each peptide–protein pair to define DRiP peptides and retiree peptides. DRiP, defective ribosomal products; H/L, heavy-to-light; HLA, human leukocyte antigen; IFNs, interferons; SILAC, stable isotope labeling by amino acids in cell culture.

To define the effect of the IFN on the DRiPome, heavy amino acid incorporation rates were followed using LC-MS/MS (dynamic SILAC method) as previously described (15, 69, 71). Two biological replicas and two technical replicates of each treatment regimen were performed. HLA peptides were declared as DRiPs or retirees based on their synthesis rates whose significance was delimited by the SDs of the synthesis rates of the tryptic peptides of each of their source proteins (see Defining Peptides as DRiPs or Retirees) (Fig. 1). Furthermore, the full proteome of MCF-7 cells of a label-free experiment, untreated or treated with IFN-β or IFN-γ for 48 h, was analyzed, to add information on the proteins affected by the IFNs. Three biological replicates of the treated label-free cells, and four biological replicas of the untreated label-free cells were analyzed.

### Antibodies

W6/32 mAbs (a mouse anti-pan-HLA-A, -B, and -C) were produced by the HB-95 hybridoma (79). The hybridoma was obtained from the American Type Culture Collection and grown in CELLine CL 350 bioreactor flasks (Integra). Rabbit polyclonal anti-HLA class I and mouse monoclonal anti-STAT1 antibodies were purchased from Abcam, mouse monoclonal anti-β1i/PSMB9 (anti-LMP-2) and mouse monoclonal anti-20S proteasome β1 antibodies from Santa Cruz, mouse monoclonal anti-actin antibody from MP Biomedicals, and peroxidase-conjugated goat anti-rabbit and goat anti-mouse IgG from Jackson ImmunoResearch.

### Immunoaffinity Purification of HLA Complexes

HLA class I molecules were purified from approximately 5 × 10^8^ cells, as previously described (80), with minor modifications, as described (71). Briefly, cells were lysed with 0.25% sodium deoxycholate, 0.2 mM iodoacetamide, 1 mM EDTA, 1:200 protease inhibitors cocktail, 1 mM PMSF, and 1% octyl-β-D-glucopyranoside (Sigma Aldrich) in PBS (4 °C, 1 h). Extracts were then cleared by centrifugation (45 min, 47,000*g*, 4 °C), and the HLA class I molecules were immunoaffinity-purified using the w6/32 mAb bound to protein A resin beads (GenScript), as previously described (71). HLA molecules with their bound peptides were then eluted with five column volumes of 1% TFA and then loaded on disposable C_18_ columns (Harvard Apparatus). The peptide fraction was recovered with 30% acetonitrile and 0.1% TFA. The peptides were dried using vacuum centrifugation, reconstituted with 100 μl 0.1% TFA, reloaded on C_18_ StageTips, prepared as previously described (81), eluted with 80% acetonitrile, dried, and then reconstituted with 0.1% formic acid for LC-MS/MS analysis. HLA peptidomes were analyzed in two biological replicas and two technical repeats (supplemental Table S1).

### Trypsin Digestion of the Proteins

Five Coomassie-stained gel slices (30-μg MCF-7 protein/lane) from each protein sample were processed by in-gel proteolysis (82). The proteins in the gel slices were reduced with 2.8 mM DTT, at 60 °C, for 30 min, and carbamidomethylated with 8.8 mM iodoacetamide in 100 mM ammonium bicarbonate, at room temperature for 30 min, and digested overnight, at 37 °C, in 10% acetonitrile and 10 mM ammonium bicarbonate with modified trypsin (Promega), at a 1:10 (wt/wt) enzyme-to-substrate ratio. The in-gel proteolysis of the labeled proteins extract was performed with two biological replicas (supplemental Table S2), while the in-gel proteolysis of the label-free proteome was performed with four biological replicas for the untreated cells and three biological replicas for the treated cells (supplemental Table S3).

### HLA Peptide and Tryptic Peptide Identification

LC-MS/MS analyses of the HLA and the tryptic peptides were performed with a Q-Exactive Plus mass spectrometer fitted either with an Easy-nLC 1000 capillary HPLC (Thermo Fisher Scientific) or with an UltiMate 3000 RSLC nano-capillary UHPLC (Thermo Fisher Scientific). Reversed-phase chromatography was performed with a homemade, 30-cm-long, 75-μm-inner diameter capillaries, packed with 3.5-μm silica ReproSil-Pur C18-AQ resin (Dr Maisch GmbH), as previously described (83). Peptides were eluted using a linear gradient of 5 to 28% acetonitrile in 0.1% formic acid, at a flow rate of 0.15 μl/min, for 2 h. Data were acquired using a data-dependent ’top 10’ method, fragmenting the peptides by higher energy collisional dissociation. Full-scan MS spectra were acquired at a resolution of 70,000, at 200 m/z, with a target value of 3 × 10^6^ ions. Fragmented masses were accumulated to an automatic gain control target value of 10^5^, with a maximum injection time of 100 msec. No fragmentation was attempted for HLA peptides with unassigned precursor charge states, or with charge states of four and above. For tryptic peptides, fragmentation was performed for charge states of 2 to 7. The peptide match option was set to Preferred. The normalized collision energy was set to 25% and MS/MS resolution was 17,500 at 200 m/z. Fragmented m/z values were dynamically excluded from further selection for 20 s.

### Data Analysis

All the LC-MS/MS data were analyzed by the MaxQuant computational proteomics platform (84), version 1.5.8.3. The database search was performed by the Andromeda search engine (85). Peptide and protein identifications were based on the April 2017 human section of the UniProt database (http://www.uniprot.org/) containing 70,946 proteins and 70,965 entries. Mass tolerances of 4.5 ppm for the precursor masses, and 20 ppm for the fragments, were allowed. Carbamidomethyl cysteine was accepted as a fixed modification for the proteomics data. Methionine sulfoxide and n-acetylation were set as variable modifications for both the proteomics and HLA peptidome analyses. Minimal peptide length was set to seven amino acids, and a maximum of two miscleavages was allowed for tryptic peptides. The false discovery rate for tryptic peptides and HLA peptides was set to 0.01 and 0.05, respectively. The MS data of the proteomics and HLA peptidomics were deposited into the ProteomeXchange (http://proteomecentral.proteomexchange.org) *via* the PRIDE partner repository (86), with the data set identifier: PXD022633.

### Data Filtration

The lists of identified and quantified HLA peptides and cellular proteins were filtered to remove reverse and potential contaminants (Fig. 1). Furthermore, peptides were assumed true HLA binders if they had a typical HLA class I ligand lengths of 8 to 14 amino acids, and the NetMHC (http://www.cbs.dtu.dk/services/NetMHC/) rank values for each of their presenting HLA alleles was ≤2 (supplemental Table S4) (87). Only proteins identified with at least two tryptic peptides were used for the next steps of the analysis. Furthermore, t-tests to determine the significance of differences of identified label-free proteins, measured in treated *versus* untreated cells, were performed by the Perseus software (88) (supplemental Table S3).

### Defining Peptides as DRiPs or Retirees

**Fig. 2:**
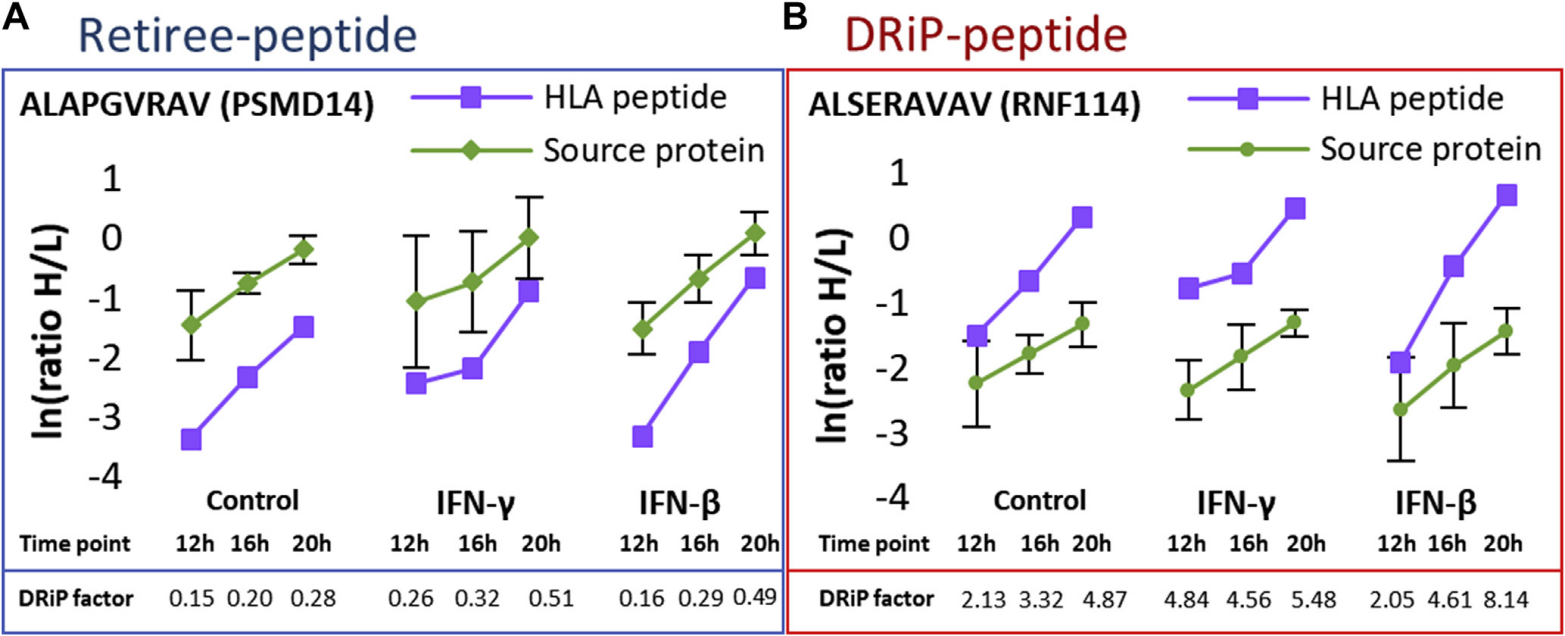
**Graphical presentation of the typical synthesis rates of retiree peptides and DRiP peptides from their corresponding source proteins.** Examples are (*A*) PSMD11 (26S proteasome non-ATPase regulatory subunit 11) and (*B*) RNF114 (E3 ubiquitin-protein ligase). The synthesis rate is observed as a shift from the light to heavy form (H/L ratio) of the HLA peptides and their corresponding source proteins. DRiP, defective ribosomal products; HLA, human leukocyte antigen.

DRiP factors are defined as the ratio between the rates of synthesis of HLA peptides and synthesis rates of their source proteins (supplemental Table S4). The relative rates of synthesis of HLA peptides or proteins were calculated from their heavy-to-light (H/L) ratios at each time point. The rates of source protein synthesis were calculated as the median H/L ratios of the different tryptic peptides at each time point. DRiP-derived HLA peptides (DRiP peptides) shift from their light to heavy forms at faster rates than their source proteins, whereas retiree-derived HLA peptides (retiree peptides) shift slower than their source proteins. In this study, peptides were classified as DRiP peptides if their H/L ratios were at least one unit higher than the SD of the H/L ratios of the tryptic peptides of their corresponding source proteins. Retiree peptides were classified as such if their H/L ratios were at least one SD lower (representative examples are shown in Fig. 2). The SD of the H/L protein ratios was calculated from the MaxQuant Ratio H/L variability [%] (as reported in the ProteinGroups.txt file in supplemental Table S4), which is the coefficient of variability of all redundant quantifiable peptides and is calculated as the SD of the natural logarithm of ratios, times 100 (84).

Part of the presented data is the result of partial H/L quantification, in which only the proteins were assigned H/L ratios, whereas their derived HLA peptides were not, and vice versa. Such pairs could therefore not be defined as classic DRiPs or retirees. Some of these HLA peptides may be presented very slowly or quickly on the cell surface, and in such cases, would only be detected in their light or heavy forms. To address this issue, the intensities (and not H/L values) of these HLA peptides were examined, and when only light or heavy intensity values were observed, they were defined as ‘extreme DRiPs’ or ‘extreme retirees’, respectively (supplemental Table S4). It should be emphasized that the classification of HLA peptides as extreme DRiPs or extreme retirees was based solely on their intensity values, rendering it less reliable than the DRiP/retiree assignments based on H/L ratios. Furthermore, it is also possible that the IFN-induced alteration of the scheme of protein synthesis in the cells caused some HLA peptides to be defined differently between the untreated and the IFN-treated cells. For example, HLA peptides, which are defined as retiree peptides in the untreated cells, were classified as DRiP peptides in the treated cells. This group of HLA peptides was termed ’transient DRiPs’ (supplemental Table S4).

## Results

To investigate the effect of IFNs on the HLA peptidome, and specifically on the DRiPome, the synthesis rates of cellular proteins and their derived HLA peptides were determined from the same culture of MCF-7 human breast cancer cells, at specified time points after IFN exposure (Fig. 1). The synthesis rates were defined by following the incorporation rates of the three heavy isotope–labeled amino acids, lysine, arginine, and leucine (see Experimental Procedures) into the cellular proteins simultaneously with their degradation products, the HLA peptides. The MCF-7 cells were cultured in growth media containing light amino acids for 8 h in the presence of 250 units of IFN-γ or 500 units of IFN-β, followed by shifting to growth media containing the heavy amino acids that were supplemented with the same concentrations of IFN-γ or IFN-β, to continue the exposure to the cytokines. Different treatment times were evaluated to select the time window where the effect of the IFNs started to be observed (8 h, supplemental Fig. S1*A*), after which, samples were taken at shorter intervals (Fig. 1) for optimal analysis of the dynamic SILAC data. The rates of protein synthesis were calculated based on the rates of incorporation of the heavy amino acids at 12, 16, and 20 h of IFNs exposure, and time intervals were selected to achieve the optimal effect of the IFNs (supplemental Fig. S1). This way, the relative rates of synthesis of the different proteins and their derived HLA peptides could be defined. In total, 12,636 HLA peptides (supplemental Table S1) and 7016 proteins (supplemental Table S2) were identified. Of these, 5189 HLA peptides derived from 2647 source proteins were assigned H/L ratios by MaxQuant (Fig. 1) and their DRiP factor values were calculated using the relative rates of synthesis of each pair of HLA peptide and its source protein (Fig. 2 and supplemental Table S4).

In theory, all tryptic peptides derived from the same protein should have similar H/L ratios at each time point, with distributions indicating small variances arising from measurement inaccuracies. In contrast, different HLA peptides derived from a single protein differ in their H/L ratios, in accordance with their stabilities and half-times while bound to the HLA molecules. The variance of the H/L ratios of the tryptic peptides of each source protein was used here to set the thresholds for declaring HLA peptides deriving from the same source protein as DRiP or retiree peptides, with at least one SD unit higher or lower, respectively (see Experimental Procedures; representative examples are shown in Fig. 2). As expected, more tryptic peptides with smaller variances between the H/L ratios were detected from the more abundant proteins, and therefore, narrower deviations of their H/L ratios could be set to define their DRiP and retiree peptides (supplemental Fig. S2). It should be emphasized that many identified HLA peptides and proteins were not defined as DRiPs or retirees because insufficient H/L ratio data were collected at certain time points and were therefore excluded from the subsequent analyses.

### IFNs Affected the DRiP More Than the Retiree Peptides

**Fig. 3:**
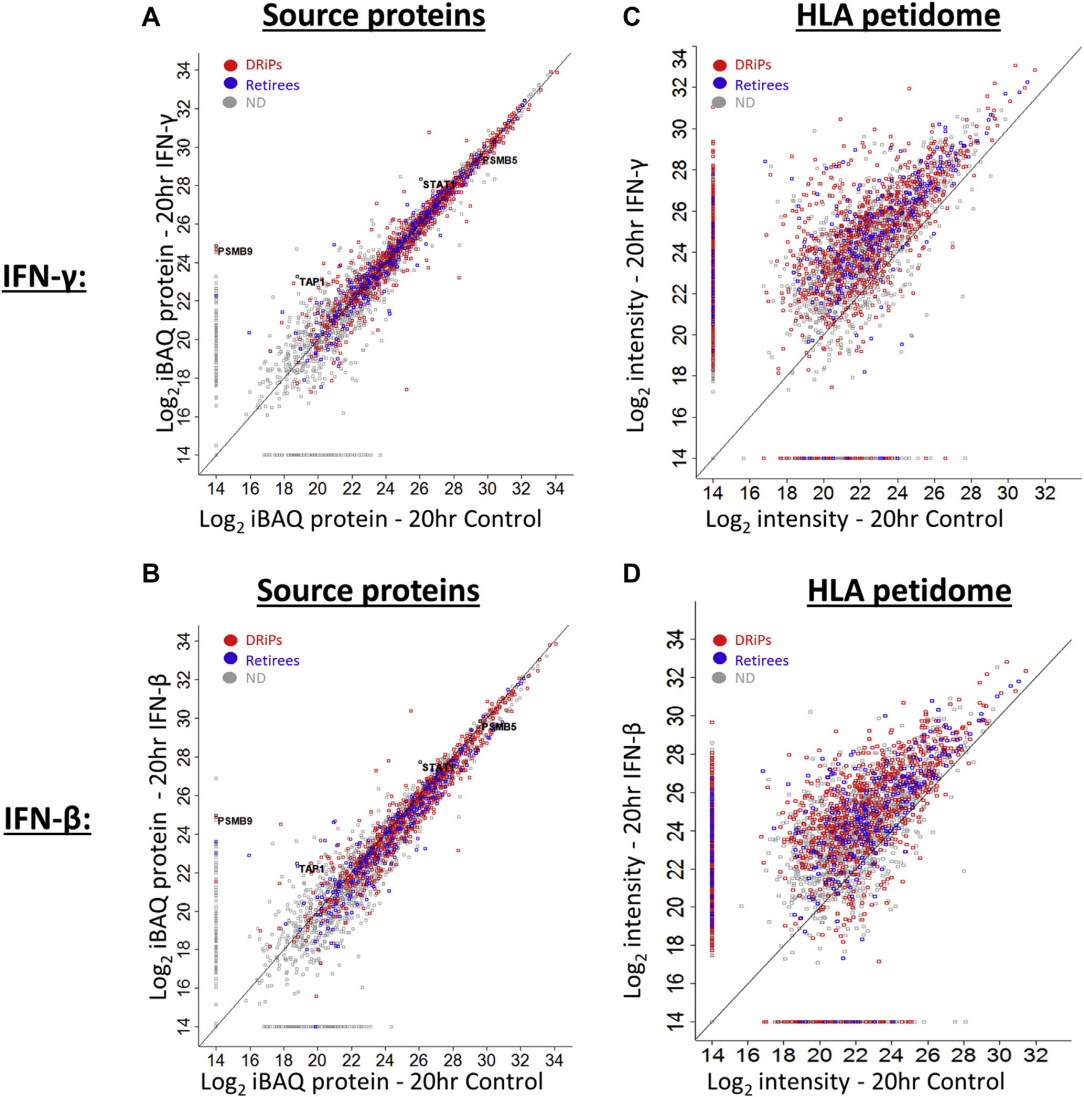
**IFN treatment upregulates the levels of DRiPs and retiree peptides and their source proteins.** The scatter plots indicate the log_2_ values based on averaged iBAQ (76) signal intensity of DRiP and retiree source proteins, before and after 20-h treatment with IFN-γ (*A*) or IFN-β (*B*). The scatter plot depicting DRiP peptides and retiree peptides indicates the LC-MS signal intensities with and without treatment with IFN-γ (*C*) or IFN-β (*D*). The DRiPs and retiree peptides are marked in *red* and *blue*, respectively; the *gray* markings represent proteins or peptides, which could not be defined (ND) as DRiPs or retirees. DRiP, defective ribosomal products; IFNs, interferons.

IFN treatment modulated both the expression levels of many proteins and their rates of synthesis (H/L ratios), along with the increase in the levels of many well-known IFN-dependent proteins, including STAT1, TAP1, and PSMB9 (Fig. 3, *A* and *B*, supplemental Fig. S1*B*, and supplemental Table S2). Similarly, as expected (23, 36, 38, 39), HLA class I molecules were upregulated by IFN treatments (supplemental Fig. S1*B* and supplemental Table S2), which was paralleled by higher intensities of HLA peptides, of which larger numbers were identified, quantified, and assigned H/L ratios (Fig. 3, *C* and *D*, Table 1, supplemental Tables S1, S3, and S4). The total number of DRiP peptides increased about twofold under these conditions, whereas the numbers and identities of most retiree peptides remained mostly unaffected (Table 1 and supplemental Table S4). Taken together, IFN exposure had a more pronounced effect on DRiPs than on retirees.

**Table 1:** The number of identified and quantified HLA peptides and proteins

Treatments	Identified HLA peptides	HLA peptides assigned H/L ratio	Identified proteins	Proteins assigned H/L ratio	HLA peptides with DRiP factors	DRiPs	Retirees
Control	3354	2527	6115	3755	1589	893	346
IFN-γ	5490	3762	6120	3680	2403	1592	253
IFN-β	5748	3675	6134	3627	2326	1312	310

Control cells were untreated.

### IFN Exposure Leads to the Presentation of DRiP Peptides With Higher DRiP Factors

**Fig. 4:**
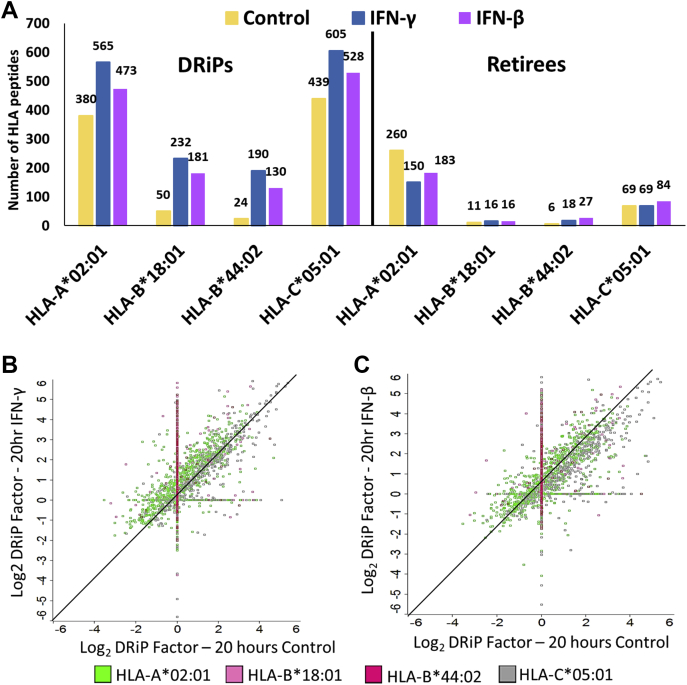
**IFNs significantly increase the presentation of DRiP peptides with high DRiP factors.** The numbers of the identified DRiP peptides and retiree peptides, which were associated with their presenting HLA allotypes, are shown (*A*). The DRiP factors of the HLA peptides presented by each of the HLA allotypes are indicated in log_2_ values, with and without 20-h treatment with IFN-γ (*B*) or IFN-β (*C*). DRiP, defective ribosomal products; HLA, human leukocyte antigen; IFNs, interferons.

Our previous study showed that IFNs upregulate the HLA-B peptidome more than the HLA-A and HLA-C peptidomes (39). To further explore if this effect influences the presentation of HLA allotype-specific DRiPs, we examined the HLA peptides presented by each of the four allotypes of the MCF-7 cells, HLA-A∗02:01, B∗18:01, B∗44:02, and C∗05:01, associated with their presenting HLA allotypes using the NetMHC server. This clustering analysis indicated that while retiree peptides presented by HLA-A, HLA-B, and HLA-C were largely unaffected by the IFN treatments, the numbers of DRiP peptides presented on HLA-B molecules were more significantly increased than the DRiP peptides presented on HLA-A and HLA-C, while the levels of their DRiP factor of both HLA-B and HLA-C were increased relative to the HLA-A peptides (Fig. 4, supplemental Fig. S3, and supplemental Table S4). One possible reason for the higher presentation of DRiP peptides after IFNs treatments could be the production of DRiP peptides with higher affinities to their presenting HLA molecules relative to the retiree peptides. However, no significant differences were observed in the predicted affinities of the DRiP peptides *versus* retiree peptides after exposure to IFNs (supplemental Fig. S4), using the affinity values defined by the NetMHC 4.0 server (87). Because more hydrophobic peptides are considered better substrates for TAP (48, 49), calculation of the grand average of hydropathy (89) using the GRAVY calculator (http://www.gravy-calculator.de/) of the DRiP and retiree peptides presented on the HLA-A, HLA-B, and HLA-C alleles did not indicate differences in the hydrophobicity of the examined peptides (supplemental Fig. S5). Thus, the increased presentation of HLA-B DRiP peptides after IFN treatment was likely not the result of a higher affinity of the HLA-B DRiP ligands to their presenting HLA-B molecules nor of their higher hydrophobicity relative to the retiree peptides.

### Surplus Subunits Induced by the IFN Treatments Are Major Contributors of Ligands to the DRiPome

**Fig. 5:**
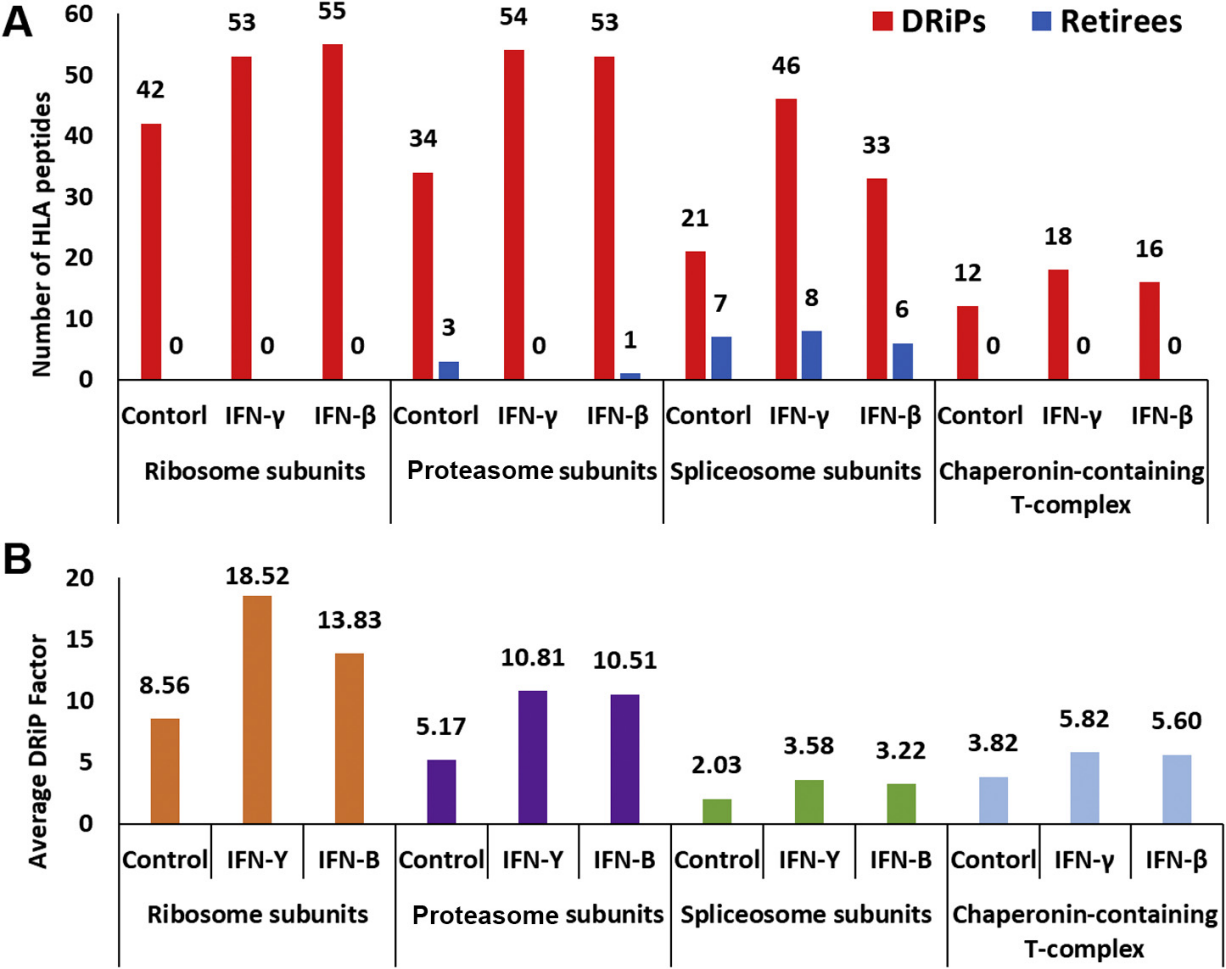
**Protein complexes contribute mostly DRiP peptides.** The number of DRiP peptides and retiree peptides deriving from different protein complexes in IFN-treated and untreated cells (*A*). The mean DRiP factors of HLA peptides in IFN-treated and untreated cells, deriving from the protein subunits of the proteasome, ribosome, spliceosome, and chaperonin-containing T complex (*B*). DRiP, defective ribosomal products; HLA, human leukocyte antigen; IFNs, interferons.

To determine whether specific cellular pathways or protein complexes contribute to the DRiPome after IFN treatments, we analyzed the lists of DRiP with the DAVID functional annotation software tool (supplemental Fig. S6 and supplemental Table S5). The analysis revealed that the ribosome, proteasome, and the chaperonin-containing T-complex (CCT) subunits contributed mostly DRiP peptides, while the spliceosome subunits contributed both retiree and DRiP peptides (Fig. 5*A*). Furthermore, after IFN treatments, the DRiP factors of the peptides deriving from the ribosome and proteasome subunits were higher than those of the spliceosome and CCT (Fig. 5*B*). Therefore, a larger focus was placed on the DRiP peptides deriving from the ribosome and proteasome protein subunits in the subsequent analyses.

### After IFN Exposure, the Standard Proteasome Contributes DRiP Peptides With Higher DRiP Factors Than the Immunoproteasome

**Fig. 6:**
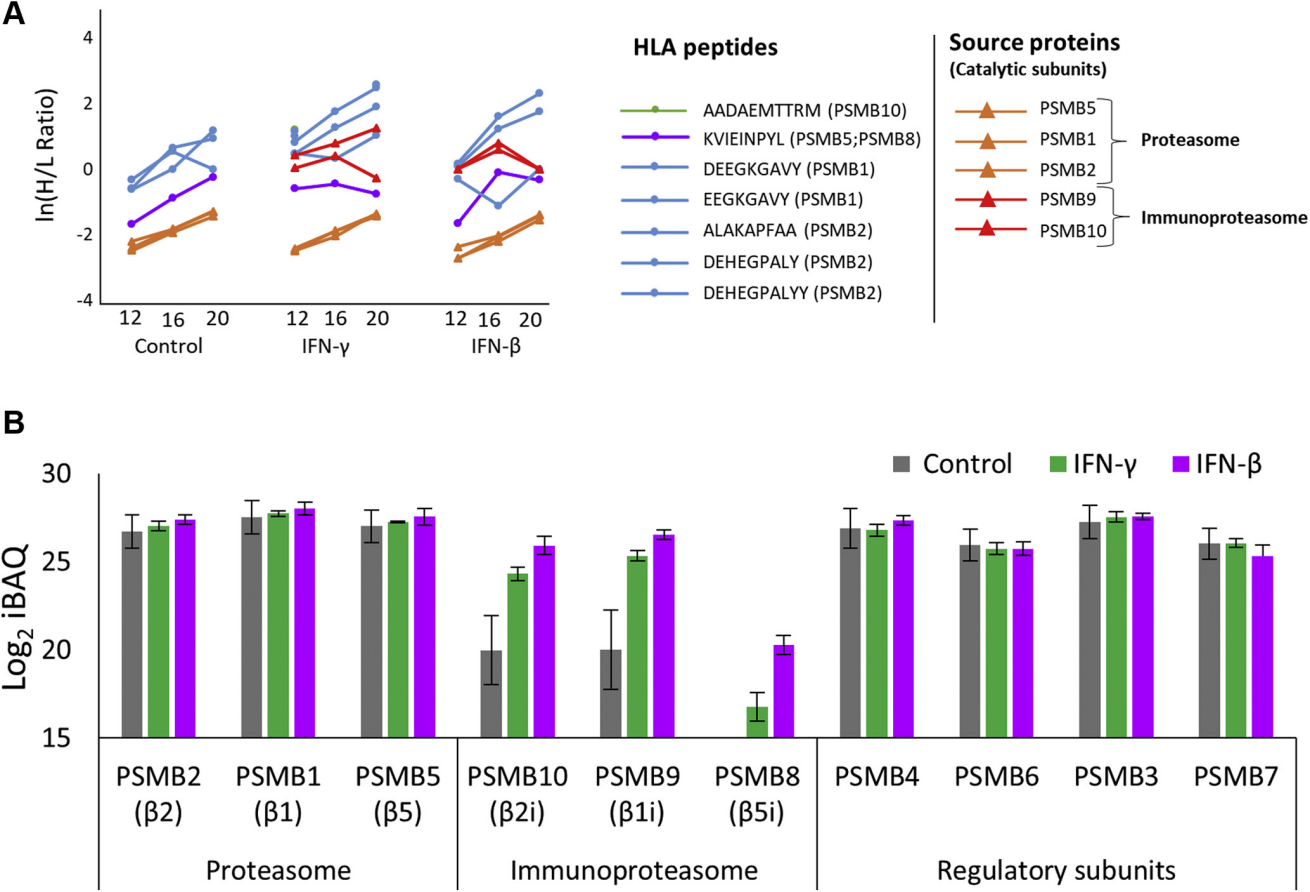
**Preferred degradation of catalytic standard proteasome subunits to DRiP peptides after IFN treatment.** The H/L ratios of the catalytic protein subunits of the standard proteasome or immunoproteasome and their derived HLA peptides are shown in ln values (*A*). The expression levels (in log_2_ iBAQ values) of the standard-proteasome and immunoproteasome subunits and their regulatory proteins needed for the assembly and functionality of both the standard proteasome and the immunoproteasome. The displayed proteomics data were of the label-free proteomics analysis after a 48-h IFN exposure (*B*). DRiP, defective ribosomal products; H/L, heavy-to-light; HLA, human leukocyte antigen; IFNs, interferons.

IFNs differentially affected the immunoproteasome relative to the standard proteasome. For example, the catalytic subunits of standard proteasome subunits were not upregulated by IFNs, whereas the catalytic subunits of the immunoproteasome were strongly upregulated (Fig. 6, *A* and *B*, supplemental Tables S2 and S3), as was observed before (48, 90). In the current analysis, five DRiP peptides deriving from the standard-proteasome catalytic subunits were detected, while the only DRiP peptide (KVIEINPYL) that could have originated from the immunoproteasome catalytic subunit PSMB8 (β5i) is also present in the parallel standard proteasome catalytic subunit PSMB5 (β5) (Fig. 6*A*), and therefore, could not be declared as a DRiP or a retiree. These observations suggest that IFN treatments induce more degradation of newly synthesized standard-proteasome catalytic subunits than of the immunoproteasome catalytic subunits, although the latter were synthesized at higher rates (Fig. 6, *A* and *B*) but barely degraded. The newly synthesized standard-proteasome catalytic subunits may have become surplus subunits and were degraded into DRiP peptides because their positions within the proteasomes were occupied by the newly synthesized immunoproteasome catalytic subunits.

To examine if other newly synthesized proteins contribute significantly to the IFN-induced DRiPome, we matched the larger proteome data of the 48-h label-free experiment to the dynamic SILAC HLA peptidome data, to evaluate the contribution of DRiP peptides derived from the downregulated proteins. Indeed, a significant enrichment of DRiP peptides deriving from mostly downregulated proteins was observed (supplemental Fig. S7, supplemental Tables S3 and S4).

### The Highest DRiP Factor Peptides Were Derived From Ribosomal Proteins

**Fig. 7:**
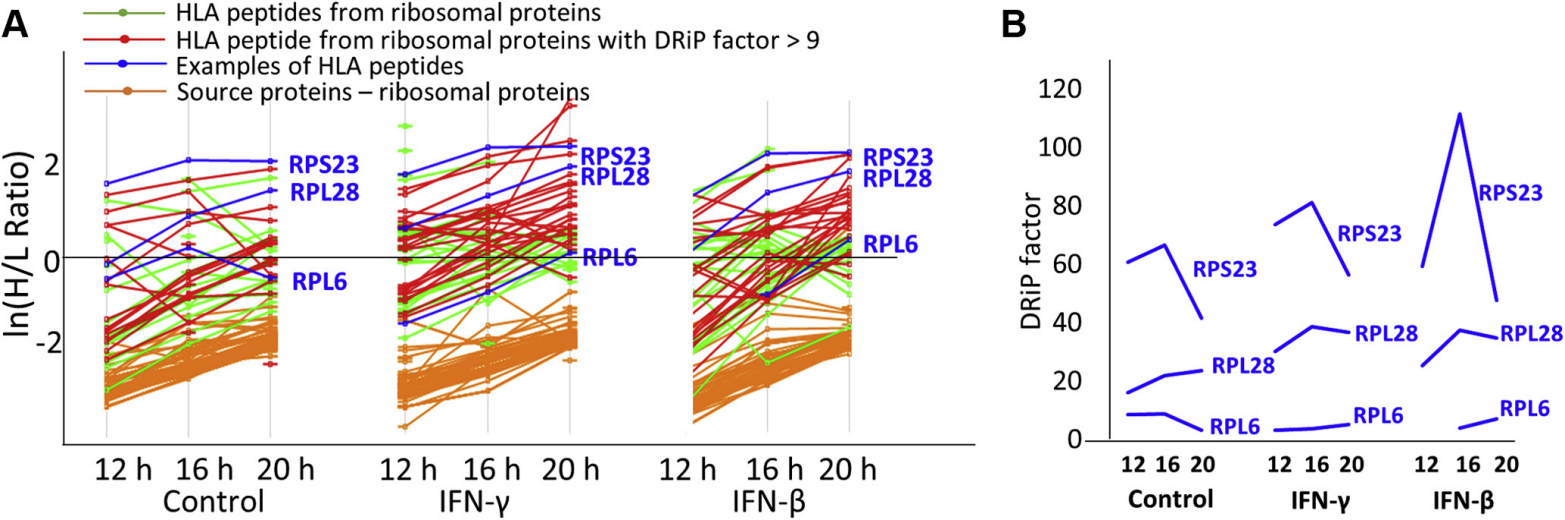
**The ribosomal protein subunits contributed DRiP peptides with higher DRiP factors after IFN exposure.***A*, the H/L ratios of the ribosome protein subunits and their derived HLA peptides are displayed in ln(H/L ratio). Labeled in *red* are HLA peptides with DRiP factors higher than the mean (>9) of the ribosomal DRiP peptides in the untreated cells (Fig. 5*B*). *B*, selected examples of ribosomal DRiP peptides (marked in *blue* in panel *A*) that increased in their DRiP factor values, after IFN treatments (RPS23, RPL28) or remain unaffected after IFN treatment (RPL6) in the 12-, 16-, and 20-h time points. DRiP, defective ribosomal products; H/L, heavy-to-light; HLA, human leukocyte antigen; IFNs, interferons.

After IFN treatments, the average DRiP factors of the DRiP peptides of the ribosomal proteins were highest among the other protein complexes examined in this study (Figs. 5*B* and 7A). The high DRiP factor ribosomal peptides were derived equally from the 40S and the 60S (Table 2 and supplemental Table S4). Interestingly, RPL28 and RPL6, which are shown here to be the source proteins of high and low DRiP factor peptides, respectively (Fig. 7), were previously shown to possess opposing roles in DRiP generation; RPL6 is thought to enhance the production of DRiP peptides, whereas RPL28 has been suggested to hamper the supply of DRiP peptides (65). Moreover, after IFN exposure, the DRiP factor of RPL28 increased, whereas the DRiP factor of RPL6 decreased (Fig. 7*B*).

**Table 2:** High DRiP factor peptides derived from ribosomal protein subunits

Source protein	HLA peptides	DRiP factor IFN-γ 20 h	DRiP factor IFN-β 20 h	Involved in ribosome biogenesis and associated with ribosomopathies^a^	Involved with viral infections
RPL10A	TLYEAVREV	9.3	7.2		(98)
	NMVAKVDEV	14.1	10.5		
	NENMVAKV	25.0	21.7		
RPL15	ILIDPFHKA	6.7	28.0	(107)	(97)
RPL24	RTDGKVFQF	13.2	12.1		
RPL26; RPL26L1	DEVQVVRGHY	17	19.7	(108)	
	DEVQVVRGH	18.1	16.1		
RPL28	AADGKGVVV	36.8	34.7		
RPL31	NEVVTREY	28.4	22.7	(109)	
RPL34	FLIEEQKIVV	10.8	15.4		
RPL35A	ALLKIEGVYA	9.6	4.0		
RPL5	KIYEGQVEV	10.9	6.5		
RPL7	MEDLIHEIY	16.6	10.4		(99)
RPS15A	VLADALKSI	7.5	13.4	(110)	
RPS16; ZNF90	YVDEASKKEI	46.2	1.3		
RPS17	ALDQEIIEV	15.2	3.7	(111, 112, 113, 114, 115)	
RPS23	DEVLVAGF	6.7	11.6	(116)	(101)
	VGDIPGVRF	56.3	47.6		
RPS3	YVDTAVRHV	66.0	51.9		(102)
RPS3A	GLKGRVFEV	8.0	11.9		(117)
	LADLQNDEV	9.2	21.3		
RPS4X	SIDKTGENF	50.3	53.4		(118)
RPS5	AETPDIKLF	4.0	16.9		
	VAETPDIKL	162.4	47.2		
RPS7	VEHKVETF	25.3	–	(111, 119, 120, 121)	(122)
RPS8	RIIDVVYNA	21.0	18.7		(123)

a Information regarding the involvement of these proteins in ribosomopathies (105).

### IFN-Induced DRiP Peptides Are Derived From the Entire Length of Their Source Proteins

It was previously proposed that DRiP peptides are preferentially derived from pioneering rounds of translations of defective mRNA transcripts, and therefore, DRiPs were suggested to be more commonly present in regions close to the N termini of their source proteins (9, 24). In contrast, DRiPs were suggested to be located evenly along the entire lengths of proteins (15). The locations of the HLA peptides, including the IFN-induced DRiP peptides and retiree peptides of the ribosome, proteasome, and spliceosome protein subunits, were evenly spread along the entire length of their source proteins, similar to the rest of the DRiP peptides identified in this study (supplemental Fig. S8 and supplemental Table S4).

## Discussion

Identification of DRiP-derived HLA peptides presentation is important for both a basic understanding of antigen presentation and for clinical immunology. The notion that MHC presentation of DRiP peptides serves as an early warning during pathogen infection was proposed before (9, 74). In our previous study (15) as well as here, using our dynamic SILAC methodology, we define DRiP peptides a bit different from the definitions proposed by Yewdell and Bennink (9, 74). The dynamic SILAC approach allows us to follow the rates of synthesis of thousands of different paired proteins and MHC peptides, at once, from the same cell culture. Thus, we can define DRiP peptides as those MHC peptides that are synthesized (become heavy) faster than their source proteins (and therefore have a high DRiP factor). This can happen only if a significant fraction of the newly synthesized protein molecules are degraded during or immediately after synthesis, and some of the degradation products are rapidly channeled to MHC presentation. In contrast, retiree proteins complete their functional lifetime and are subsequently degraded; thus, their MHC peptides shift from their light to heavy forms slower than their source proteins. It should be emphasized that this approach enables the definition of DRiPs and retirees that are derived from both fast and slow turning over proteins. The DRiPs are the fraction of protein molecules that are degraded before the proteins mature, and the retirees are the fraction of protein molecules that are degraded after the proteins completed their functional lifetimes, irrespective if they are derived from fast or slow turning over proteins. However, some HLA peptides are derived from very rapidly degrading proteins, which are often observed only in their heavy forms, and therefore are called here ‘extreme DRiPs’ and are listed in supplemental Table S4. At the other end, ‘extreme retirees’ are HLA peptides that were detected only in their light forms in this study. Yet, it should be emphasized that the inability to detect the light or heavy forms of some peptides may be caused just by a failure of the bioinformatics software and should be further investigated elsewhere.

The major role of IFNs in coping with viral infections is well documented (91); however, the detailed involvement of IFNs in the modulation of the repertoire presentation of DRiPs has not been studied before, although their roles in enhancing the production of DRiPs from ubiquitinylated, oxidized, and partially unfolded proteins were studied before (23). The IFN enhancement of HLA presentation (38, 44) and altering cellular metabolism and gene expression (31, 92) are also well studied. The IFNs may enhance the presentation of DRiP peptides to enhance the alerting of the immune system about ongoing infections. IFNs induce the expression of some proteins, while blocking the expression, and even inducing the degradation of others (Figs. 6 and 7, supplemental Fig. S7, supplemental Tables S2 and S4). When viruses infect cells, the cells respond by expressing type-I IFNs and by inducing the expression of type-II IFN-γ by immune cells (31). A well-documented consequence of elevated IFN levels is the increased expression of MHC and presentation and upregulation of chaperones and enzymes involved in antigen processing and presentation (2, 4, 7, 45). These observations indicate that IFNs serve as possible triggers of protein degradation (23), a phenomenon that may extend to faster degradation of viral and surplus subunits of cellular protein complexes that are left unassembled because of the IFN response and subsequent recruitment of the immune system. The examples studied here included the proteasomes (Fig. 6 and supplemental Table S4) and the ribosomes (Fig. 7 and supplemental Table S4). IFNs regulate the expression and activities of different proteins by altering posttranslational modifications (23, 93) and by synthesis or degradation of specific subunits (31). Identification of alterations in the subunit compositions of protein complexes was followed through isolation of protein complexes or full-proteome analyses while looking for deviations from the stoichiometry of these subunits (76, 77, 94). However, following the degradation schemes of the surplus subunits before they are incorporated into the protein complexes is very challenging because the degradation process is often too rapid to be followed through proteolysis intermediates. The current work overcame some of these hurdles by combining MS-based proteomics and MHC peptidomics technologies with dynamic SILAC, which enabled large-scale analysis of the degradation of these proteins through their easily detectable proteolysis products, that is, DRiP or retiree MHC peptides (15, 69, 71). Yet, even this high-throughput dynamic SILAC methodology may be biased to the detection of DRiP peptides from more abundant protein and peptides that are more rapidly synthesized, and more rapidly degraded, and therefore can be detected in both their light and heavy isotope forms above the threshold limits of the mass spectrometer.

Previous studies on the HLA presentation of DRiP-derived peptides have monitored selected DRiP peptides (65) and even the entire DRiPome of untreated cells (15, 69, 71). These studies concluded that indeed DRiPs constitute a significant part of the HLA peptidome. Although the effects of IFNs on the HLA peptidome were studied in several large-scale analyses (39, 58, 59, 60, 61, 62, 63), this work is the first to follow the effect of IFNs on the entire DRiPome by combining dynamic SILAC with large-scale proteomics and HLA peptidomics analyses.

The presented findings demonstrated that IFN exposure had a major effect on the DRiPome, as manifested by the induced presentation of numerous new DRiP peptides, alongside only minor effects on the presentation of retiree peptides (Fig. 4*A*, and Table 1). This may be the result of enrichment of the peptide ‘pool’ in the peptide-loading compartment of the ER with degradation products of defective newly synthesized proteins. Alternatively, DRiPs may be preferentially loaded on the MHC molecules in a dedicated pathway or by specific IFN-regulated chaperones. However, because both DRiP peptides and retiree peptides are loaded on the same MHC alleles, it is unclear how the chaperones could distinguish between the two. Compartmentalized loading of DRiP ligands *versus* retiree ligands may also underlie the observed shift in their relative distributions (95). The presented experimental techniques were unable to distinguish between loading occurring in different cellular compartments, and further research is needed to resolve this question. Interestingly, groups of extreme DRiPs from very fast degrading proteins and extreme-retiree peptides from very slow degrading proteins (supplemental Table S4) were noted in the dynamic SILAC analysis. The extreme DRiPs and extreme retirees detected here did not seem to derive from specific cellular pathways. The same applied to the group of transient DRiPs, which are HLA peptides identified as DRiPs or retirees in the IFN-treated cells differently from the untreated cells (supplemental Table S4).

We have previously suggested that many DRiP peptides are disproportionally derived from surplus subunits of protein complexes, such as the ribosome and CCT, in untreated cells (15). Such surplus subunits are likely produced in excess of their requirement for assembly of a protein complex and are subsequently rapidly degraded. In IFN-treated or virus-infected cells, such a phenomenon may be a side product of the IFN response and may reflect a situation in which viral proteins are left unincorporated into the virions and are subsequently degraded and presented by the MHC. Alternatively, the IFNs may induce the degradation of subsets of proteins as part of their regulation of the cellular metabolism and facilitation of responding to viral infection. The data presented here suggest that IFNs induce the expression of surplus orphan subunit products, including some from the proteasomes and the ribosomes (supplemental Table S4), even in the absence of viral infection. A good example of presentation of HLA peptides derived from surplus subunits is the presentation of standard-proteasome DRiP peptides, observed here when the IFN-induced newly expressed subunits of the immunoproteasome are preferred for incorporation into the mature proteasomes leaving the corresponding standards proteasomes subunits unassembled and directed for degradation (Fig. 6*A* and supplemental Table S3). The fact that no HLA peptides (including DRiPs or retirees) deriving from the immunoproteasome subunits were identified, despite their increased expression following IFNs treatment (Fig. 6*A* and supplemental Table S4), suggests that the preferential incorporation of the immunoproteasome subunits into the proteasome scaffold (96) hardly leaves these subunits as surplus unincorporated proteins for degradation, whereas it leaves a surplus of standard-proteasome subunits that continue to be synthesized after the IFN treatment (Fig. 6) because of inefficient shutdown of their synthesis but cannot be incorporated into the proteasomes and need to be degraded. It should be emphasized that both proteasome isoforms are expressed in the cells during inflammation, with the immunoproteasome estimated to constitute then up to 60% of the total proteasome population (43). The standard-proteasome subunits targeted for degradation are likely not defective but are excess functional proteins and therefore may be more appropriately referred to as orphan subunit products rather than DRiPs. Although there was a low expression level of immunoproteasomes in the untreated cells (Fig. 6), close to half of the DRiP peptides were still detected in the untreated cells (Table 1 and supplemental Table S4), suggesting that the proteolytic activities of both the standard proteasome and the immunoproteasome contribute similarly to the production of the DRiP peptides. In addition, the observation that downregulated proteins, due to the IFN treatment, contribute excessively to the DRiPome suggests that induction of active degradation of some proteins by IFN treatments (supplemental Fig. S7) is part of the cellular response mechanism to the viral infection and IFN responses. Further studies are needed to address this issue.

In untreated cells, the ribosomal DRiP peptides were associated with high DRiP factors, relative to the other protein complexes observed in this work (Fig. 5*B*). IFN exposure elevated (by about twofold) the DRiP factor values of the ribosomal DRiP peptides, whereas no retiree peptides were identified among the HLA peptides deriving from ribosomal proteins (Fig. 5*B* and supplemental Table S4). While the effects of IFNs on ribosome subunits have been less studied, the interactions of both 40S and 60S ribosomal proteins with viruses have been studied extensively and determined to be important in the translation of viral components needed for the assembly of new virions (97, 98, 99, 100, 101, 102). Modulation of ribosomal protein synthesis and assembly in response to viral infection (77, 94) provides a mechanism for preventing the assembly of the 80S ribosome, which subsequently curbs the synthesis of viral proteins. Another possibility is that during viral infection, cells produce special types of ribosomes, called immunoribosomes, with different subunit compositions (103, 104). It is possible that IFNs also induce expression of such immunoribosomes and therefore the unneeded subunits are degraded and these immunoribosomes enhance the DRiP production and the immune responses. In this study, both the 40S and 60S ribosomal subunits were similarly represented among the high-DRiP-factor DRiPome in response to the IFNs (supplemental Table S4), possibly as part of the cellular scheme of limiting ribosomal function during viral infection. Interestingly, two of the ribosomal proteins shown here to be sources of DRiP peptides (RPL28 and RPL6) were also shown before to be involved with DRiP enhancement and ubiquitin-dependent MHC peptide presentation (65). The observation in this study may indicate that these subunits are misassembled into immunoribosomes after IFN treatment, and subsequently degraded. In addition, about a third of the ribosomal DRiP peptides identified here are derived from subunits known to interact with viruses and to be involved with the virus infectious cycle (Table 2). Most of the ribosomal DRiP peptides had high DRiP factors, possibly because their source protein subunits are quickly degraded after IFN exposure to cope with the viral infection. Other ribosomal proteins associated with high DRiP peptides, identified in our study (Table 2), are not known to interact with viruses. Interestingly, high DRiP factor DRiP peptides (Table 2) were also derived from proteins known to be associated with ribosomopathies (105). It should be mentioned that ribosomal protein composition is dynamic and complex, allowing for ribosome specialization, *via* translation regulation and adaptation to stress (77, 94, 106). Further studies are needed to better understand the involvement of IFNs in the modulation of ribosomal protein subunits and subsequent synthesis of DRiP peptides, and their role in the cellular responses to viral infections.

## Data Availability

The mass spectrometry data of the HLA peptidome and proteomes (labeled and label-free) have been deposited to the ProteomeXchange (http://proteomecentral.proteomexchange.org) *via* the PRIDE partner repository (86), with the data set identifier: PXD022633.

## Supplemental data

This article contains supplemental data (87).

## Conflict of interest

The authors declare no competing interests.
