# Supplementary material for: The Effect of Interferons on Presentation of Defective Ribosomal Products as HLA Peptides
Source: Mol Cell Proteomics. 2021 Jun 1;20:100105. doi: 10.1016/j.mcpro.2021.100105 (PMC8724922; doi:10.1016/j.mcpro.2021.100105)
Supplement: Supplemental Figures S1–S8 [file mmc1.pdf]

Supplemental Material

The effect of interferons on presentation of defective ribosome products as HLA peptides

Authors: Liran Komov ‡, Dganit Melamed Kadosh ‡, Eilon Barnea, and Arie Admon

‡ Authors contributed equally

List of contents:

- Supplemental Tables S1-S5 legends
- Supplemental Figures S1-S8

Supplemental Tables:

Table S1: MaxQuant output of the HLA peptides identified and quantified in the Dynamic SILAC experiment (peptides.txt).

Table S2: MaxQuant output of the cellular proteome quantified in the Dynamic SILAC experiment (protein groups.txt)

Table S3: MaxQuant output of the cellular proteome quantified in the label-free experiment after a 48 h exposure to IFNs (protein groups.txt).

Table S4: Data of filtered HLA peptides, matched with their source proteins. The data listed in the table were filtered, as detailed in Figure 1. DRiP-factors of pairs of HLA-peptide and their source proteins are shown, as well as their classification as DRiP-peptides or retiree-peptides. The extreme DRiP-peptides, retiree-peptides, and transient DRiP-peptides are listed as well.

Table S5: Results of the DAVID functional annotation analysis. The list contains annotations of DRiPs-peptide and retirees-peptide source proteins.

Supplemental Figures:

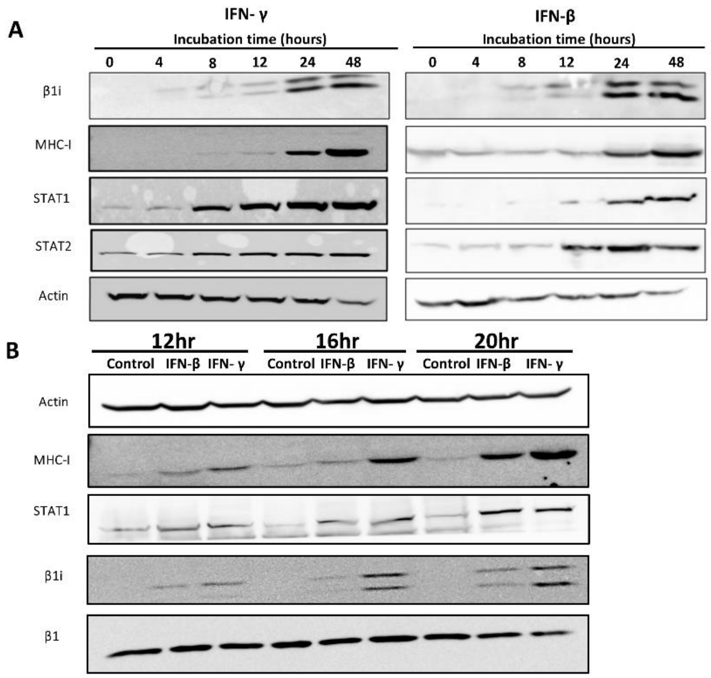

Figure S1: The effect of IFN-β and IFN-γ on the expression of IFN-regulated proteins.

(A) The effects of IFNs on HLA class I molecules, STAT1, STAT2, and the immunoproteasome subunit β1i (PSNB9), after treatment with IFN-β or IFN-γ for 4, 8, 12, 24, and 48 h. Proteins were analyzed by western blot of total cell extracts. (B) The effects of IFNs on HLA class I molecules, STAT1, standard proteasome subunit β1 (PSMB1) and the immunoproteasome subunit β1i (PSMB9), after treatment with IFN- β or IFN-γ for 12, 16 and 20 h. Proteins were analyzed by western blot of total cell extracts taken from the dynamic SILAC experiment.

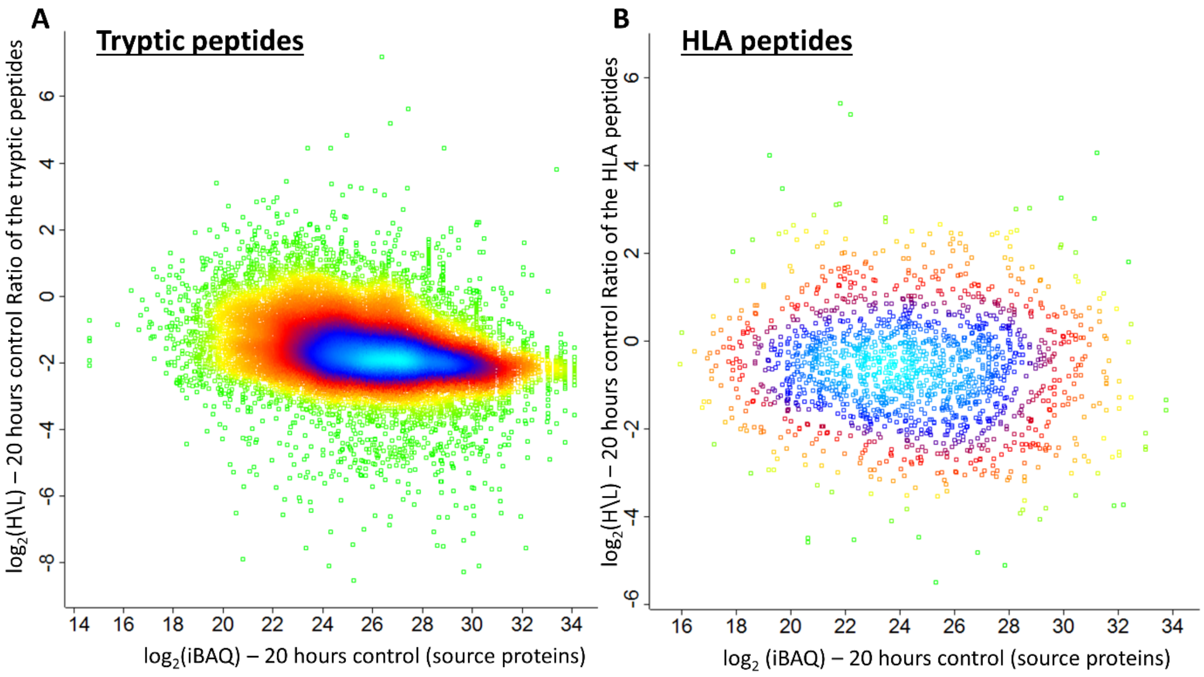

Figure S2: Lower variance in the H/L ratios of tryptic peptides relative to HLA peptides. (A) The scatter of $\log_2$(H/L ratios) of tryptic peptides and HLA peptides (B) from each source protein are shown relative to the expression levels of their source proteins in $\log_2$(iBAQ) values.

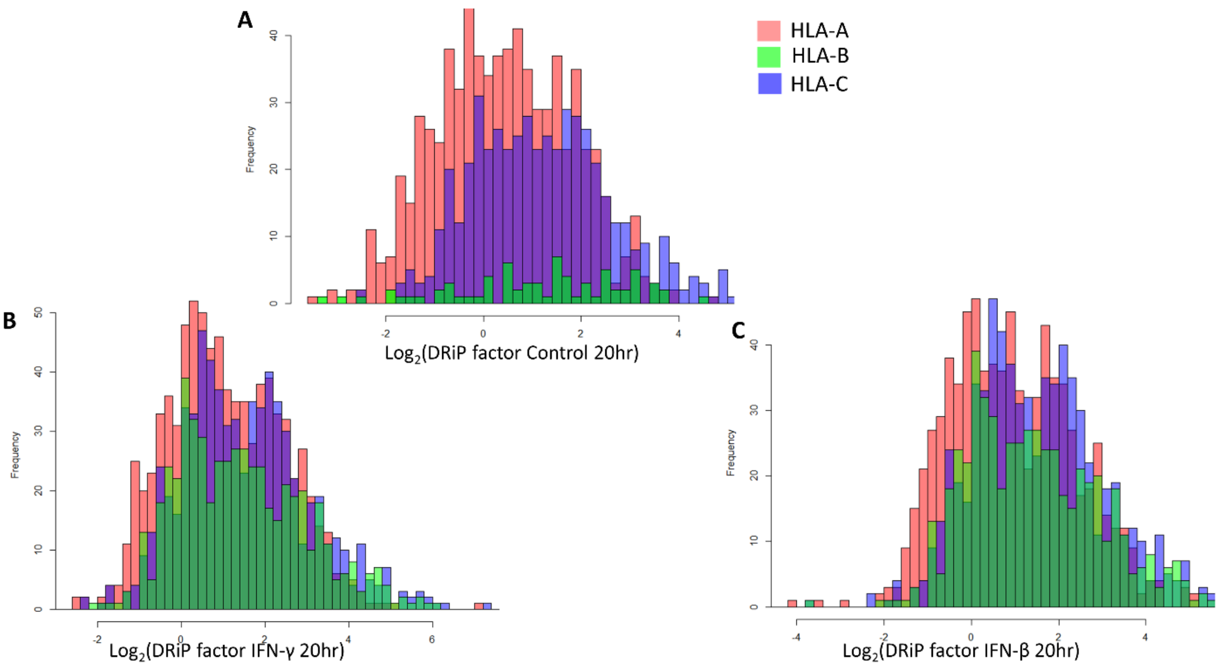

Figure S3: DRiP-factors of HLA-B and HLA-C peptides were higher than HLA-A peptides following IFNs exposure. The distribution of DRiP-factors of 20 h IFN-treated cells and untreated cells is displayed in $\log_2$ values. (A) Untreated cells; (B) IFN-γ-treated cells; (C) IFN-β-treated cells. The HLA-peptides presented on HLA-A*02:01 molecules are marked in pink, the HLA-B*18/B*44 in green, and HLA-C*05 in blue.

Figure S4

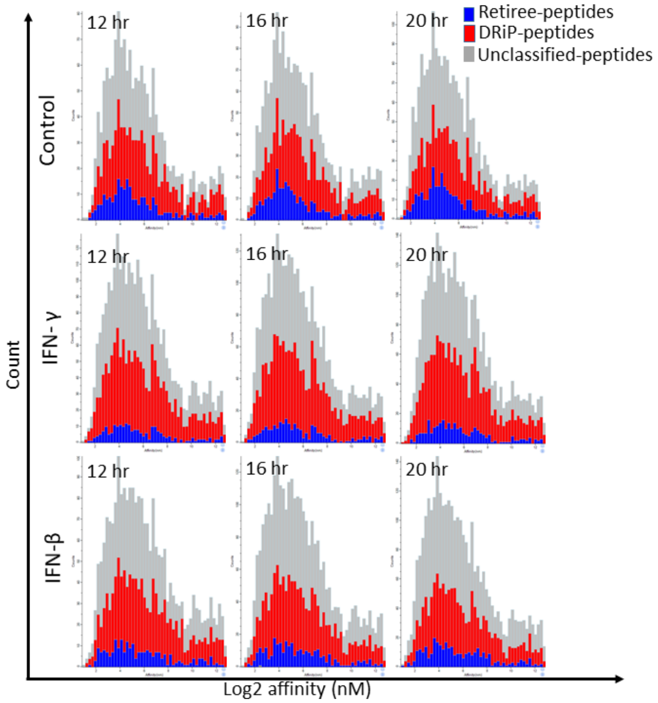

Figure S4: The affinities of the presented DRiP-peptides and retiree-peptides are similar. The predicted affinities of DRiP, retiree, and unclassified peptides to their presenting HLA molecules at the different time points, were calculated by the NetMHC server. The affinity values were only calculated for HLA peptides with rank ≤ 2, which is the acceptable NetMHC limit for MHC binders (Andreatta and Nielsen, 2016).

GRAVY values histograms

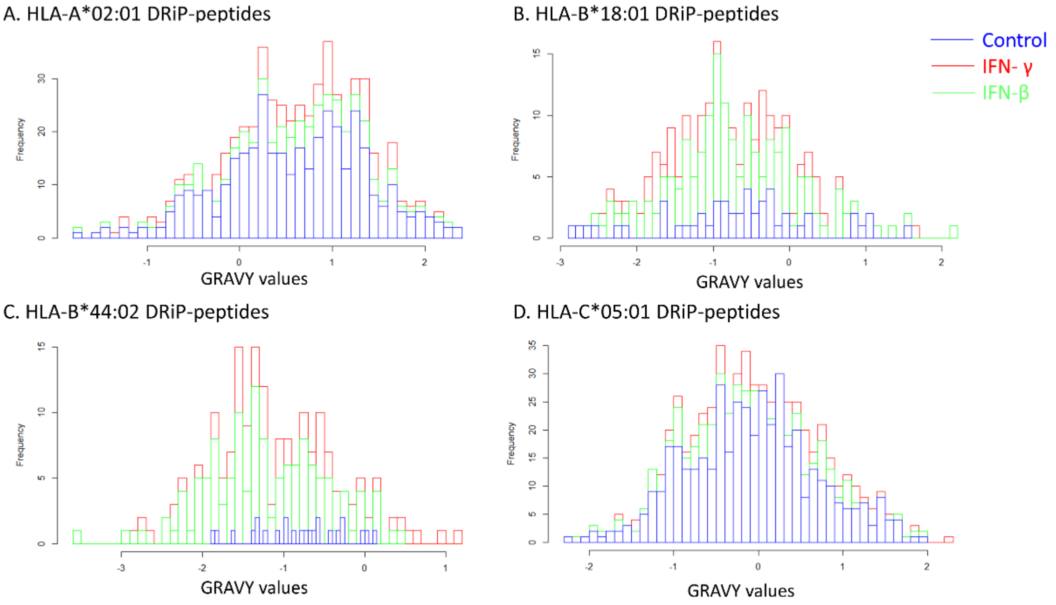

GRAVY values histograms

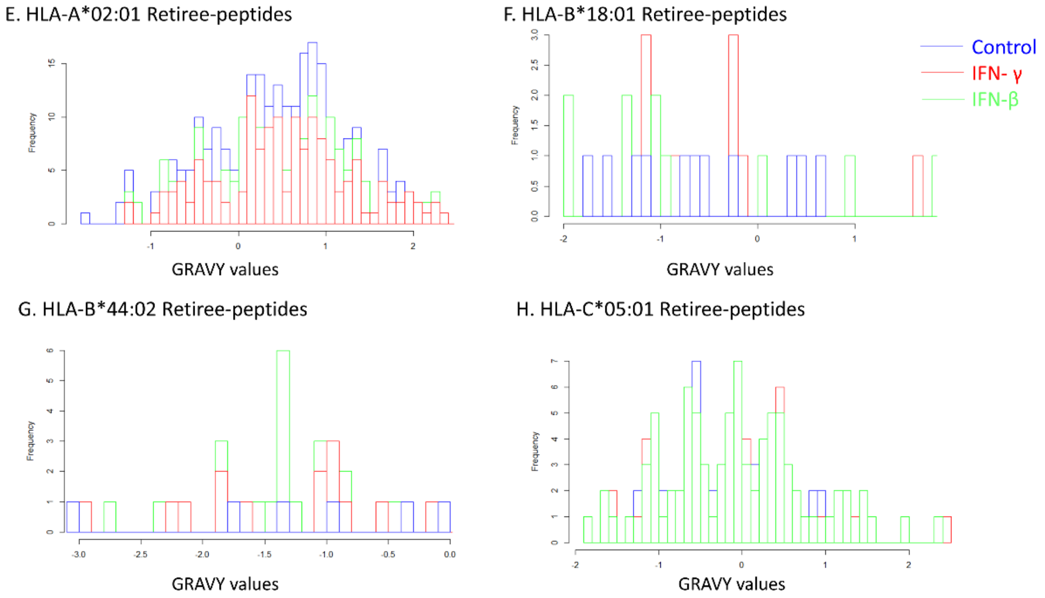

Figure S5: The hydrophobicity values of the presented DRiP-peptides are similar to those of the retiree-peptides. The GRAVY values of DRiP-peptides (A-D) and retiree-peptides (E-H) presented on the HLA molecules: HLA-A*02:01 (A, E), HLA-B*18:01 (B, F), HLA-B*44:02 (C, G), or HLA-C*05:01 (D, H) were calculated using the GRAVY calculator platform.

Enrichment annotation analysis by DAVID Bioinformatics Resources 6.8

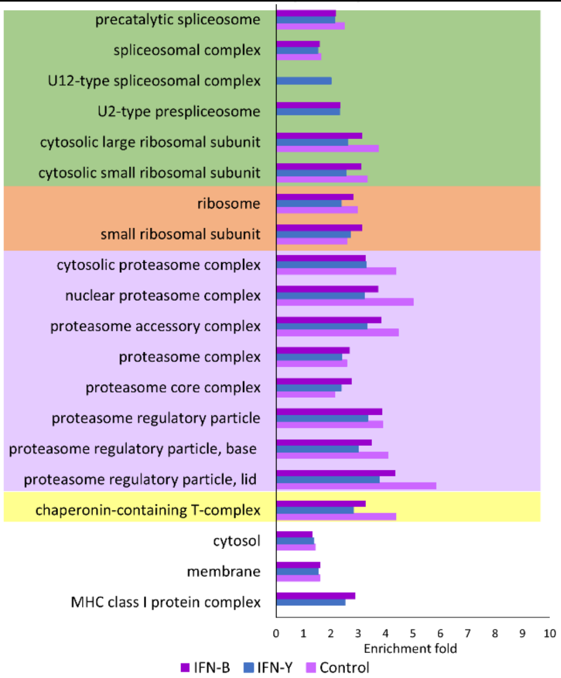

Figure S6: The IFN treatments affected more DRiP-peptides derived from protein complexes. The list of the source protein of the DRiP-peptides was analyzed by the DAVID functional annotation tool. The enrichment results are listed in Table S5. The spliceosome components are highlighted in green, ribosome in orange, proteasome in purple, and chaperonin-containing T-complex in yellow.

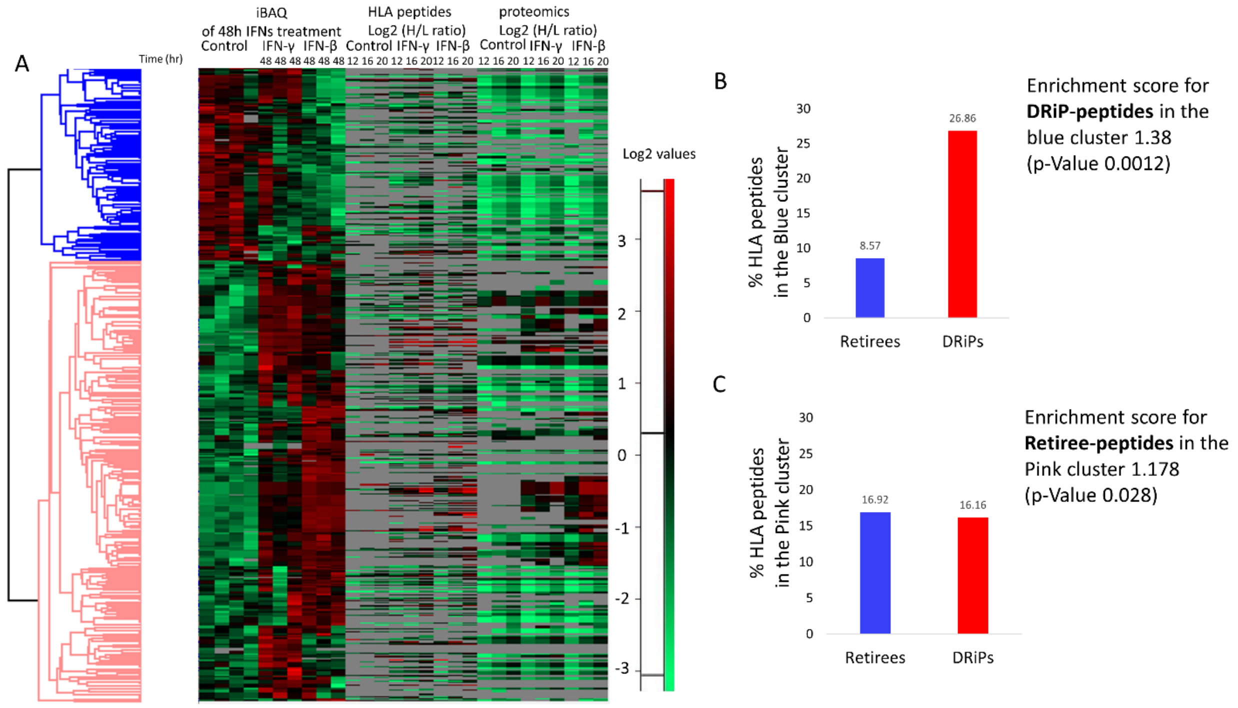

Figure S7: DRiP-peptides are preferentially derived from IFN-downregulated proteins.

(A) The heatmap is divided into clusters of proteins downregulated (blue cluster) and upregulated (pink cluster) following IFN treatments. The heatmap is divided into clusters based on the label-free proteomics data, collected after the 48 hours exposure to IFNs (left side). Added to the heatmap are the HLA peptidome (middle) and proteomics data (right side) of the dynamic SILAC experiment, at the 12, 16, and 20 hours time points. Enrichment scores are of DRiP-peptides belonging to downregulated proteins (B) or of retiree-peptides belonging to upregulated proteins (C) following IFNs treatments.

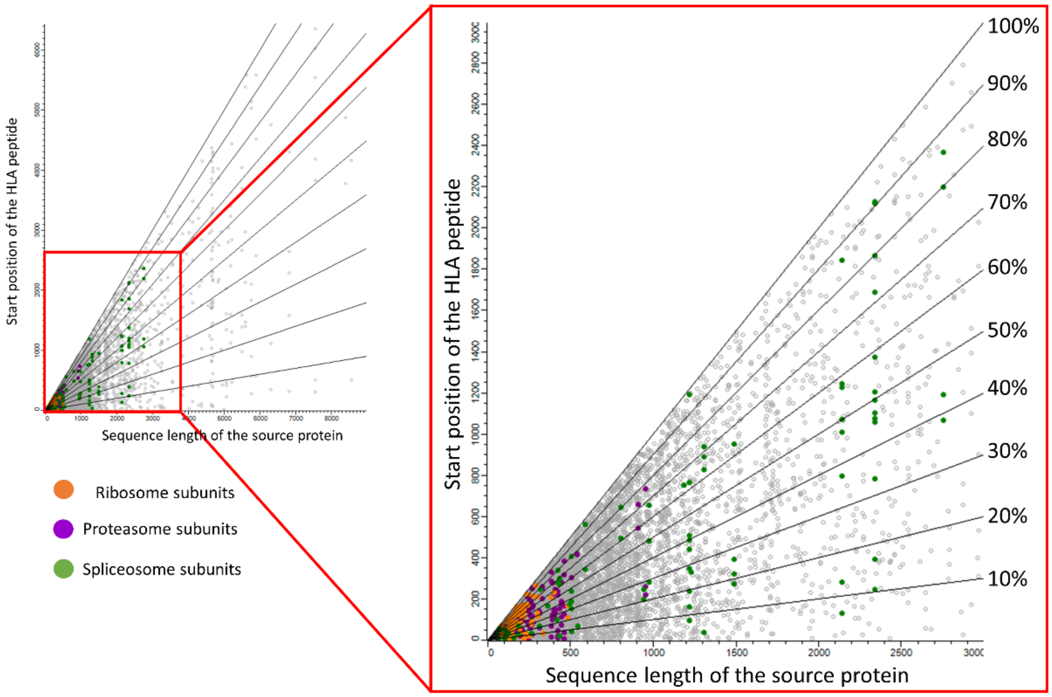

Figure S8: Relative location of HLA peptides within their source proteins. The colored dots represent HLA-peptides deriving from the ribosome (orange), proteasome (purple), and spliceosome (green) protein subunits, while the gray dots represent the remaining HLA-peptides and their source proteins. The diagonal lines represent the percentile values of the locations of the HLA peptides within their source proteins.
